# Supplementary material for: Lytic and Latent Genetic Diversity of the Epstein–Barr Virus Reveals Raji-Related Variants from Southeastern Brazil Associated with Recombination Markers
Source: Int J Mol Sci. 2024 May 3;25(9):5002. doi: 10.3390/ijms25095002 (PMC11084898; doi:10.3390/ijms25095002)
Supplement: Supplementary file 1 [file ijms-25-05002-s001.zip › ijms-2967882-supplementary.pdf]

Supplementary Material

Lytic and latent genetic diversity of the Epstein–Barr virus reveals Raji–related variants from southeastern Brazil associated with recombination markers

Paula Alves*, Paulo Rohan, Rocio Hassan, Eliana Abdelhay

1 Supplementary Tables

Table S1 - Sampling characteristics

ID isolate	Donor type	Collection year	Tissue type	*BZLF1* phylogeny	*LMP1* phylogeny (Alves et al. 2022)	EBV type	Zp
ACBR02	asymptomatic carrier	2018	saliva	BZ-B	Raji	T1	V1
ACBR03	asymptomatic carrier	2018	saliva	BZ-A	Raji	T2	V3
ACBR04	asymptomatic carrier	2018	saliva	BZ-B	Mediterranean	T1	V1
ACBR05	asymptomatic carrier	2018	saliva	BZ-A	Unclassified	T2	V3
ACBR07	asymptomatic carrier	2018	saliva	BZ-B	B95-8	T1	V1
ACBR08	asymptomatic carrier	2018	saliva	BZ-B	Mediterranean	T1	V1
ACBR12	asymptomatic carrier	2018	saliva	BZ-B	Unclassified	T1	V1
ACBR13	asymptomatic carrier	2018	saliva	BZ-A	Unclassified	T2	V3
ACBR14	asymptomatic carrier	2018	saliva	BZ-B	Raji	T1	V1
ACBR15	asymptomatic carrier	2018	saliva	BZ-B	Raji	T1	V1
ACBR16	asymptomatic carrier	2018	saliva	BZ-B	Raji	T1	V1
ACBR17	asymptomatic carrier	2018	saliva	BZ-B	Mediterranean	T1	V1
ACBR18	asymptomatic carrier	2018	saliva	BZ-B	Mediterranean	T1	V1
ACBR22	asymptomatic carrier	2018	saliva	BZ-A	Raji	T2	V3
ACBR23	asymptomatic carrier	2018	saliva	BZ-B	Mediterranean	T1	V1
ACBR24	asymptomatic carrier	2018	saliva	BZ-A	Raji	T1	V3
ACBR25	asymptomatic carrier	2018	saliva	BZ-A	Mediterranean	T2	V3
ACBR26	asymptomatic carrier	2018	saliva	BZ-B	Mediterranean	T1	V1

ACBR27	asymptomatic carrier	2018	saliva	BZ-A	Mediterranean	T2	V3
ACBR28	asymptomatic carrier	2018	saliva	BZ-B	Raji	T1	V1
ACBR29	asymptomatic carrier	2018	saliva	BZ-A	Mediterranean	T2	V3
ACBR30	asymptomatic carrier	2018	saliva	BZ-B	China2	T1	V3
ACBR31	asymptomatic carrier	2018	saliva	BZ-A	Raji	T2	V3
ACBR32	asymptomatic carrier	2018	saliva	BZ-B	Unclassified	T1	V1
ACBR33	asymptomatic carrier	2018	saliva	BZ-B	Mediterranean	T1	V1
ACBR34	asymptomatic carrier	2018	saliva	BZ-B	Mediterranean	T1	V1
ACBR35	asymptomatic carrier	2018	saliva	BZ-A	Unclassified	T2	V3
ACBR36	asymptomatic carrier	2018	saliva	BZ-B	Mediterranean	T1	V1
ACBR37	asymptomatic carrier	2019	saliva	BZ-B	Mediterranean	T1	V1
ACBR39	asymptomatic carrier	2019	saliva	BZ-B	Mediterranean	T1	V1
ACBR40	asymptomatic carrier	2019	saliva	BZ-B	Mediterranean	T1	V1
ACBR42	asymptomatic carrier	2019	saliva	BZ-B	Mediterranean	T1	V1
ACBR43	asymptomatic carrier	2019	saliva	BZ-B	Mediterranean	T1	V1
ACBR44	asymptomatic carrier	2019	saliva	BZ-A	B95-8	T2	V3
ACBR46	asymptomatic carrier	2018	saliva	BZ-A	n/a	T2	V3
ACBR47	asymptomatic carrier	2018	saliva	BZ-B	n/a	T1	n/a
ACBR48	asymptomatic carrier	2018	saliva	BZ-B	n/a	T1	n/a
ACBR49	asymptomatic carrier	2018	saliva	BZ-B	n/a	T1	V1
ACBR50	asymptomatic carrier	2018	saliva	BZ-B	n/a	T1	n/a
ACBR51	asymptomatic carrier	2018	saliva	BZ-B	n/a	T1	V1
ACBR52	asymptomatic carrier	2018	saliva	BZ-B	n/a	T1	V1
ACBR53	asymptomatic carrier	2018	saliva	BZ-A	n/a	T2	V3
ACBR54	asymptomatic carrier	2018	saliva	BZ-B	n/a	T1	V1
ACBR55	asymptomatic carrier	2019	saliva	BZ-A	n/a	T1	V3
ACBR56	asymptomatic carrier	2019	saliva	BZ-B	n/a	T1	V1
BLBR01	Burkitt lymphoma	1995	lymph node	BZ-B	Mediterranean	T1	V1
BLBR02	Burkitt lymphoma	2001	lymph node	BZ-B	Mediterranean	T1	V1
BLBR03	Burkitt lymphoma	2002	lymph node	BZ-B	Mediterranean	T1	V1

BLBR04	Burkitt lymphoma	2003	lymph node	BZ-B	Raji	T1	V1
BLBR05	Burkitt lymphoma	2019	lymph node	BZ-B	Raji	T1	V1
BLBR06	Burkitt lymphoma	2005	lymph node	BZ-B	Raji	T1	V1
CHLBR01	classic Hodgkin lymphoma	2002	lymph node	BZ-B	Mediterranean	T1	V1
CHLBR02	classic Hodgkin lymphoma	2002	lymph node	BZ-B	B95-8	T1	V1
CHLBR04	classic Hodgkin lymphoma	2002	lymph node	BZ-B	Raji	T1	V1
CHLBR05	classic Hodgkin lymphoma	2002	lymph node	BZ-B	Mediterranean	T1	V1
CHLBR06	classic Hodgkin lymphoma	2002	lymph node	BZ-A	B95-8	T2	V3
CHLBR08	classic Hodgkin lymphoma	2003	lymph node	BZ-A	Raji	T1	V3
CHLBR09	classic Hodgkin lymphoma	2003	lymph node	BZ-B	Mediterranean	T1	V1
CHLBR10	classic Hodgkin lymphoma	2003	lymph node	BZ-A	Raji	T1	V3
CHLBR15	classic Hodgkin lymphoma	2004	lymph node	BZ-B	Raji	T1	V1
CHLBR18	classic Hodgkin lymphoma	2005	lymph node	BZ-A	Raji	T2	V3
CHLBR23	classic Hodgkin lymphoma	2005	lymph node	BZ-B	Mediterranean	T1	V1
CHLBR24	classic Hodgkin lymphoma	2005	lymph node	BZ-B	Mediterranean	T1	V1
CHLBR26	classic Hodgkin lymphoma	2005	lymph node	BZ-A	Raji	T1	n/a
PTER01	post-transplant EBV reactivation	2019	peripheral blood	BZ-B	n/a	T1	V1
PTER02	post-transplant EBV reactivation	2005	peripheral blood	BZ-B	n/a	n/a	V1
PTER03	post-transplant EBV reactivation	2005	peripheral blood	BZ-B	n/a	T1	V1
PTER04	post-transplant EBV reactivation	2005	peripheral blood	BZ-B	n/a	T1	V1
PTER05	post-transplant EBV reactivation	2006	peripheral blood	BZ-B	n/a	T1	V1
PTER06	post-transplant EBV reactivation	2006	peripheral blood	BZ-B	n/a	T1	V1

n/a: not applicable.

Table S2 - List of the geographic reference sequences used in phylogenetics analysis.

ID	Database	Access number	Country/Region origin
B95-8	GenBank	NC_007605	USA
AG876	GenBank	DQ279927	Ghana
GD1	GenBank	AY961628	China
GD2	GenBank	HQ020558	China
Raji	GenBank	KF717093	Nigeria
Jijoye	GenBank	LN827800	Nigeria
Mutu	GenBank	KC207814	Kenya
Daudi	GenBank	LN827545	Kenya
HKNPC1	GenBank	JQ009376	Hong Kong
M81	GenBank	KF373730	Hong Kong
Akata	GenBank	KC207813	Japan
Weweak2	GenBank	LN827544	Papua New Guinea
BL37	GenBank	LN827526	Africa
M-ABA	GenBank	LN827527	Africa
GK_BL42	GenBank	MG298834	Africa
GK_BL44	GenBank	MG298835	Africa
GK_BL60	GenBank	MG298836	Africa
GK_PUT	GenBank	MG298842	Africa
L591	GenBank	LN827523	Germany
DF_Tonsil_T49	GenBank	MG298829	Argentina
DF_Tonsil_T47	GenBank	MG298828	Argentina
CV-ARG	GenBank	KR063343	Argentina
VA	GenBank	KT001102	Argentina
SG	GenBank	KT001103	Argentina
sLCL-IS1.13	GenBank	LN827578	Australia
sLCL-IS1.07	GenBank	LN827594	Australia
sLCL-IM1.16	GenBank	LN827799	Australia
SCL	GenBank	KP968259	Brazil

VGO	GenBank	KP968260	Brazil
CCH	GenBank	KP968257	Brazil
MP	GenBank	KP968258	Brazil
RPF	GenBank	KR063344	Brazil
FNR	GenBank	KR063345	Brazil
C666-1	GenBank	LN827525	China
HLT011	GenBank	MK540263	China
HS007	GenBank	MK540266	China
HS027	GenBank	MK540282	China
NKLT002	GenBank	MK540303	China
NPCT098	GenBank	MK540453	China
YCCEL1	GenBank	LN827561	South Korea
pLCL-TRL1-pre	GenBank	LN824207	USA
pLCL-TRL595	GenBank	LN827559	USA
GK_Farage	GenBank	MG298839	USA
JC_002	GenBank	MG298864	USA
GK_BL67	GenBank	MG298837	France
IMS_Saliva_177	GenBank	MG298849	India
JM_LCL_MU	GenBank	MG298874	Indonesia
JM_NKTLY_96.1	GenBank	MG298877	Indonesia
JM_NPC_bru_377	GenBank	MG298885	Indonesia
RK_LCL_L2	GenBank	MG298912	Papua New Guinea
RK_LCL_L3	GenBank	MG298914	Papua New Guinea
sLCL-1.19	GenBank	LN827562	Kenya
sLCL-1.24	GenBank	LN827568	Kenya
sLCL-BL1.20	GenBank	LN827571	Kenya
sLCL-2.21	GenBank	LN827587	Kenya
sLCL-2.22	GenBank	LN831023	Kenya
GK_Akuba	GenBank	MG298830	Kenya
HL09	GenBank	LN827522	United Kingdom
HL11	GenBank	LN827524	United Kingdom
IMS_Saliva_10	GenBank	MG298844	United Kingdom

IMS_Saliva_70	GenBank	MG298861	United Kingdom
IMS_Saliva_71	GenBank	MG298862	United Kingdom
AH_Saliva_8192	GenBank	MG298823	Taiwan
AH_Saliva_8471	GenBank	MG298824	Taiwan
AH_Saliva_9077	GenBank	MG298826	Taiwan
GK_LY47	GenBank	MG298840	Uganda
JM_Saliva_18	GenBank	MG298899	Uganda
JM_Saliva_20	GenBank	MG298900	Uganda
JM_Saliva_33	GenBank	MG298901	Uganda
JM_Saliva_5	GenBank	MG298902	Uganda

Table S3: List of recombination-inducing DNA motifs evaluated in this work.

Recombination-inducing DNA motif	Motif	Reference
core chi-like recombination sequence	TGGTGG	Chuzhanova et al. 2009
classical meiotic recombination motif	CCTCCCCT	Myers et al. 2005
initiate recombination events	TGGAG<br>CCCAG	Bengesser et al. 2010
human Ig class switch sequence	GGGCT	Chuzhanova et al. 2009
arrest DNA synthesis by DNA polymerase α	AGGAG	Cullen et al. 2002

T: thymine; G: guanine; A: adenine; C: cytosine.
